# Mitochondrial mutation m.1555A>G as a risk factor for failed newborn hearing screening in a large cohort of preterm infants

**DOI:** 10.1186/1471-2431-14-210

**Published:** 2014-08-26

**Authors:** Wolfgang Göpel, Sandra Berkowski, Michael Preuss, Andreas Ziegler, Helmut Küster, Ursula Felderhoff-Müser, Ludwig Gortner, Michael Mögel, Christoph Härtel, Egbert Herting

**Affiliations:** 1 Department of Paediatrics, University of Lübeck, University Hospital of Schleswig Holstein, Ratzeburger Allee 160, G-23538 Lübeck, Germany; 2 Institute for Medical Biometry and Statistics, University of Lübeck, Lübeck, Germany; 3 Center for Clinical Trials, University of Lübeck, Lübeck, Germany; 4 Department of Paediatrics, Georg-August University, Göttingen, Germany; 5 Department of Paediatrics, Essen University Hospital, Essen, Germany; 6 Department of Paediatrics, University of Homburg, Homburg, Germany; 7 Department of Paediatrics, University Hospital Carl Gustav Carus, Dresden, Germany

**Keywords:** Newborn, Screening, Hearing loss, Mitochondrial, Mutation

## Abstract

**Background:**

The mitochondrial m.1555A>G mutation is associated with a high rate of permanent hearing loss, if aminoglycosides are given. Preterm infants have an increased risk of permanent hearing loss and are frequently treated with aminoglycoside antibiotics.

**Methods:**

We genotyped preterm infants with a birth weight below 1500 grams who were prospectively enrolled in a large cohort study for the m.1555A>G mutation. Treatment with aminoglycoside antibiotics in combination with mitochondrial m.1555A>G mutation was tested as a predictor for failed hearing screening at discharge in a multivariate logistic regression analysis.

**Results:**

7056 infants were genotyped and analysed. Low birth weight was the most significant predictor of failed hearing screening (p = 7.3 × 10^-10^). 12 infants (0.2%) had the m.1555A>G-mutation. In a multivariable logistic regression analysis, the combination of aminoglycoside treatment with m.1555A>G-carrier status was associated with failed hearing screening (p = 0.0058). However, only 3 out of 10 preterm m.1555A>G-carriers who were exposed to aminoglycosides failed hearing screening. The m.1555A>G-mutation was detected in all mothers of m.1555A>G-positive children, but in none of 2993 maternal DNA-samples of m.1555A>G-negative infants.

**Conclusion:**

Antenatal screening for the m.1555A>G mutation by maternal genotyping of pregnant women with preterm labour might be a reasonable approach to identify infants who are at increased risk for permanent hearing loss. Additional studies are needed to estimate the relevance of cofactors like aminoglycoside plasma levels and birth weight and the amount of preterm m.1555A>G-carriers with permanent hearing loss.

## Background

In 1993 a maternally transmitted non-syndromic deafness with susceptibility to aminoglycoside treatment was reported to be due to a single nucleotide (A to G) substitution at position 1555 of the mitochondrial genome [1]. The variant m.1555A>G genotype is found about 1 in 500 Europeans [2]. Subsequent studies showed that the effect of this mutation is observed, even when aminoglycoside levels are within the therapeutic range [3,4]. The m.1555A>G-mutation is frequently found in families with maternally transmitted hearing loss. In these families, all carriers of the mutation who received aminoglycosides became deaf [5]. However, in population based cohort studies hearing in children and adults carrying the mutation was not impaired [2,6].

The role of the m.1555A>G mutation has not been investigated in populations with high aminoglycoside treatment rates and routine hearing tests. We therefore screened a large cohort of preterm infants with a birth weight below 1500 grams for the m.1555A>G mutation to test, if established risk factors for congential hearing impairment and m.1555A>G in combination with aminoglycoside treatment are associated with a higher failure rate of newborn hearing screening. We furthermore screened a large number of mothers of these infants to determine if maternal genetic screening for m.1555A>G would be feasible in order to prevent postnatal aminoglycoside treatment of infants carrying the mutation.

## Methods

### Study population

The German Neonatal Network (GNN) is a prospective multicentre cohort study of preterm infants with a birth weight below 1500 grams which is supported by the German Federal Ministry of Research and Education. Improvement of the long term outcome of preterm infants is the major aim of the GNN. Some additional information is given at http://www.vlbw.de. We analysed infants with a gestational age below 37 + 0 weeks and a birth weight less than 1500 grams and their mothers who were enrolled from 46 German neonatal intensive care units between 2003 and 2012. Antenatal and postnatal clinical data were documented on standardised data sheets during the hospital course of the patients. Data quality was ensured by regular on-site-monitoring done by physicians of the central office of the German Neonatal Network at the University of Lübeck. Clinical data were coded and entered into a central database. Results of newborn hearing screening were collected during the whole study. Written informed consent for sample and data collection was given by parents of all participating infants. The study was approved by the ethics committee of the University of Lübeck and by ethics committees of all participating hospitals and universities.

### Outcomes and exposures

According to a recent review on risk factors for hearing loss in very low birth weight infants [7] and a report on vancomycin ototoxicity in neonates [8], we defined birth weight, bronchopulmonary dysplasia (BPD, defined as need for oxygen supplementation at 36 weeks post-menstrual gestational age) and treatment with furosemide, glycopeptide antibiotics or ganciclovir/valganciclovir as risk factors for failed newborn hearing screening. We used gancyclovir/valgancyclovir treatment as a marker for congenital cytomegalovirus (CMV) infection since this is the only indication for gancyclovir treatment of preterm infants. The m.1555A>G genotype in combination with documented aminoglycoside treatment was analyzed as a risk factor for failed neonatal hearing screening. Neonatal hearing screening was performed using otoacoustic emissions, usually before discharge of the infant, but some centres used brainstem evoked response audiometry.

### Genotyping

DNA was collected by buccal swabs (mothers and infants) or umbilical cord tissue (infants). DNA was extracted by standard procedures (Qiagen blood and tissue kits, Germany) and stored at -20 degrees centigrade at the University of Lübeck. DNA-samples of infants who were born between 2003 and 2012 and were available at our lab on December 31st 2012 were genotyped for m.1555A>G. We used a custom assay (TaqMan-assay, forward primer GACATTTAACTAAACCCTACGCATT, reverse primer GTCCAAGTGCACTTTCCAGTACA reporter ATGTTACGACTTGTCTCCTC / ACGACTTGCCTCCTC) for allelic discrimination with a HT7900 thermocycler (Applied Biosystems, California, USA). Maternal samples were genotyped for infants carrying the m.1555A>G-genotype and additional maternal samples of infants who had no m.1555A>G-mutation.

### Statistical methods

Statistical analysis was done with SPSS Version 20 (IBM, New York, USA), by using a logistic regression modell with failed newborn hearing screening as the dependent and birth weight (100 g – increments), any treatment with glycopeptide antibiotics, any treatment with furosemide, CMV-infection, bronchopulmonary dysplasia and aminoglycoside treatment × m.1555A>G-genotype as independent variables. The global significance level was set to 0.05. Adjustments for multiple testing were done by Bonferroni correction for multiple comparisons with 7 independent variables, and the nominal p-value thus was 0.007. All p-values are two-sided. Data are reported according to STROBE statement [9].

## Results

DNA-samples of 8504 infants were available. Infants without neonatal hearing screening (n = 892, 10.5%), or missing data for confounding variables (n = 143, 1.7%) were excluded. 412 infants were excluded because of failed m.1555A>G-genotyping and one infant because of suspected DNA-contamination with a positive control (total n = 413 infants, 4.9%). 7056 infants were finally included in our analysis.

**Figure 1:**
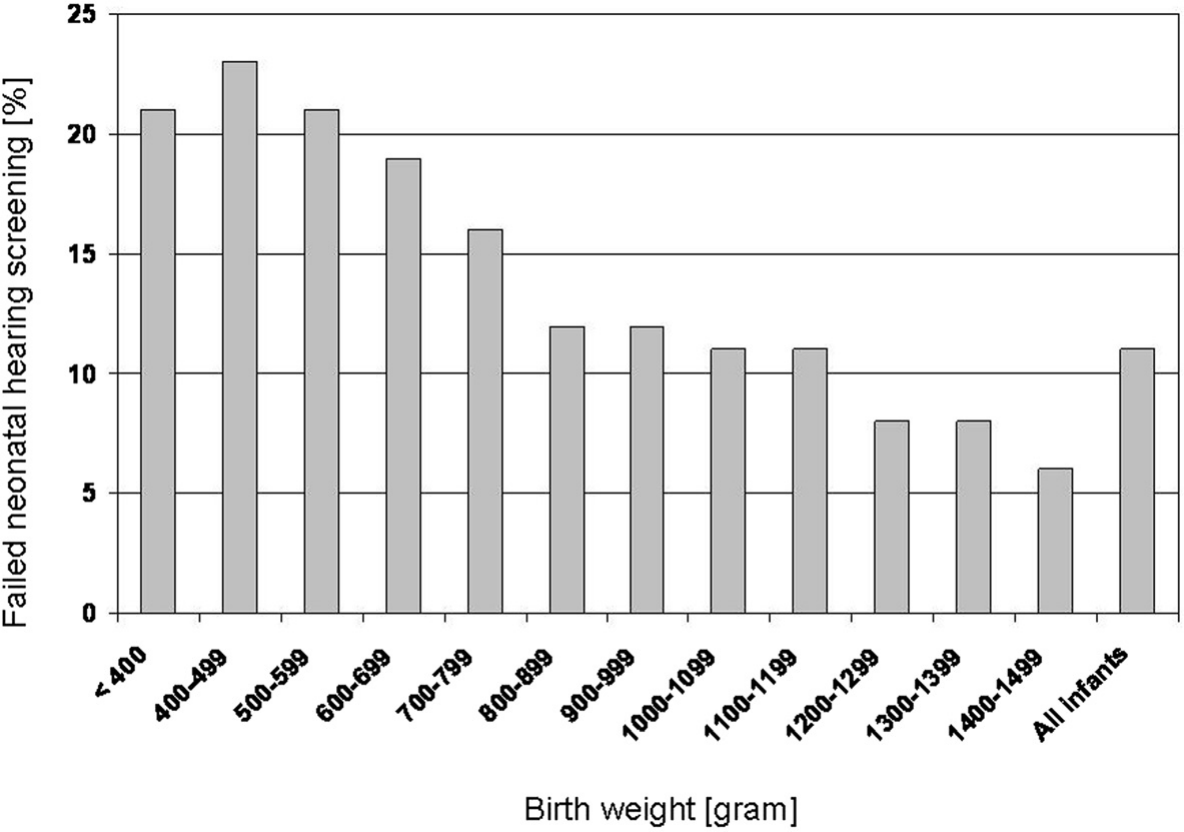
**Failed newborn hearing screening as a function of birth weight (n = 7056).** The number of infants in the < 400 g strata is 39. All other strata include > 100 infants.

3554 infants were male (50.4%) and 2357 infants (33.4%) were multiples. Clinical data and results of the multivariate logistic regression analysis are given in Table 1. Low birth weight was the most important predictor of failed hearing screening (OR 0.91, 95%CI 0.88-0.94 per 100 g additional birth weight, Figure 1).

**Table 1:** Failed neonatal hearing screening: multivariate logistic regression of risk factors

	**Failed neonatal hearing screening [% (n failed hearing screening/n with risk factor)**	**OR**	**95% CI**	**p**
All	11% (788/7056)	Ref.		
Birth weight^#^		0.91	0.88-0.94	7.3 × 10^-10^*
No Aminoglycosides, m.1555A>G -	8.5% (224/2636)	1.26	1.07-1.49	0.0058*
No Aminoglycosides m.1555A>G +	0% (0/2)			
Aminoglycosides m.1555A>G -	12.7% (561/4408)			
Aminoglycosides m.1555A>G +	30% (3/10)			
Vancomycin treatment	15.8% (349/2208)	1.24	1.04-1.47	0.015
Furosemide treatment	17.6% (238/1355)	1.29	1.08-1.57	0.0066*
Cytomegalovirus-infection	27% (6/22)	1.80	0.69-4.7	0.23
Infants with BPD	18.6% (195/1048)	1.21	0.99-1.49	0.067

^#^OR for birth weight is given per 100 g increased weight. Percentage of failed hearing screening for each category is given in Figure 1. *p < 0.007, multivariate logistic regression.

12 infants (0.2%) carried the m.1555A>G-mutation. Only 2 of these infants were not treated with aminoglycoside antibiotics like gentamicin or tobramycine. Both had normal newborn hearing screening. Three of ten m.1555A>G carriers, who were treated with aminoglycosides failed newborn hearing screening. The combination of aminoglycoside treatment × m.1555A>G-genotype was a significant predictor of failure (OR 1.26, 95% CI 1.07-1.49, p = 0.0058). If aminoglycoside treatment and m.1555A>G-genotype were analysed as independent risk factors in the same multivariate logistic regression model, none of them was predictive for failed hearing screening (data not shown). Furosemide treatment was associated with failed hearing screening as well, whereas other factors such as vancomycin treatment, cytomegalovirus-infection and BPD were not predictive. To test if other potentially important confounders are associated with hearing loss, we added sepsis (confirmed by positive blood-culture) and abnormal head scans (intra-ventricular haemorrhage detected by ultrasound) to our multivariate logistic regression model. However, both variables were not predictive for failed hearing screening (sepsis: OR 0.97, 95% CI: 0.78-1.2; p = 0.8; intra-ventricular haemorrhage: OR 0.94, 95% CI: 0.77-1.15, p = 0.5).

Additional clinical data of infants carrying the m.1555A>G-mutation are given in Table 2. Six out of twelve infants were monozygotic twin pairs. Remarkably, two twin pairs who carried the mutation had discordant hearing screening, with pathological results in the smaller infant. Genotyping of maternal DNA was successful in 3002 of 3070 samples (97.7%). The m.1555A>G-mutation was found in all 9 mothers whose infants also carried the m.1555A>G-mutation (3 twin-mothers and 6 singleton mothers). All other maternal samples were negative for m.1555A>G.

**Table 2:** Clinical data of infants carrying the m.1555A>G-genotype

**Number**	**Birth weight category [grams]**	**Gestational age [weeks]**	**Gender [m/f]**	**Mutliple [y/n]**	**Gentamicin treatment [y/n]**	**Result of hearing screening**
1	1100-1199	28	M	Y	Y	Pass
2	1100-1199	28	M	Y	Y	Pass
3	800-899	25	F	N	Y	Pass
4	1200-1299	29	M	N	N	Pass
5	1200-1299	32	F	N	N	Pass
6	1200-1299	29	F	Y	Y	Pass
7	800-899	29	F	Y	Y	Fail
8	1400-1499	32	M	N	Y	Pass
9	1300-1399	30	M	N	Y	Pass
10	1400-1499	30	F	Y	Y	Pass
11	1300-1399	30	F	Y	Y	Fail
12	<400	24	M	N	Y	Fail

Birth weight and gestational age are given as completed 100 grams/completed weeks, respectively. All aminoglycoside treated infants received gentamicin. Patients 1 and 2, 6 and 7 and patients 10 and 11 were twin pairs. None of the patients was treated with furosemide.

## Discussion

Aminoglycoside treatment is standard of care for suspected sepsis in newborns. Analysis of large scale epidemiological data indicate, that substitution of aminoglycosides with broad spectrum antibiotics such as cephalosporins as first line treatment of newborn sepsis is associated with an increased rate of death [10,11]. Therefore, general avoidance of aminoglycoside treatment of newborns may not be feasible or reasonable. However, it might be beneficial to change aminoglycoside treatment in some selected patients with a high risk of side effects, such as carriers of the m.1555A>G-mutation. Ealy et al. screened 703 neonatal intensive care unit patients and identified two m.1555A>G carriers who were both treated with gentamicin and had normal newborn hearing screening. These infants had a gestational age of 33 and 34 weeks, respectively. Birth weight was not reported [12]. Johnson et al. screened 436 infants and identified 3 infants with m.1555A>G who were treated with gentamicin. One of these infants had repeatedly abnormal hearing tests [13]. Taken together, these studies indicate, that about 20% (one of five) infants who are carriers of m.1555A>G and received aminoglycosides had abnormal hearing screening. In our study this rate was even higher (3 of 10 infants), and comparable to the failure rate of infants with CMV-infection which is an established risk factor for permanent hearing loss.

Other risk factors which were included in our analysis were associated with an increased risk of failed neonatal hearing screening as well. Low birth weight itself was the most important predictor, but all other reported risk factors were associated with higher failure rates of newborn hearing screening, with significant p-values for treatment with furosemide. As reversible hearing loss after furosemide treatment is well described, this observation might be of minor importance for current treatment [7]. However, since many preterm infants are treated with more than one ototoxic drug simultaneously, ongoing cohort studies and randomised trials involving aminoglycosides, vancomycin and furosemide should consider the combined effect of these drugs on permanent hearing loss.

Although we screened more than 7000 preterm infants, the total number of m.1555A>G carriers in our study was low, which limits the statistical power of our observation. The rate of failed hearing screening in our cohort was 11%, which is close to reported data in the literature [14]. It is important to note, that most infants with failed newborn hearing screening do not develop permanent hearing loss.

However, family studies of m.1555A>G carriers indicate a very high penetrance of permanent hearing loss if aminoglycosides are given [5], and a recently published follow up study including 19 Finish m.1555A>G carriers reported that all children passed the newborn hearing screening, but 10 of 19 developed permanent hearing loss at a median age of 3.7 years [15]. Therefore, our current data might considerably underestimate the long term effect of the aminoglycoside use in preterm infants carrying m.1555A>G.

5-year follow-up for GNN-infants who were enrolled in 2009 or later will start in 2014. Since measurement of evoked otoacoustic emissions are part of the follow-up, we will be able to analyse more data concerning the long-term effects of aminoglycoside treatment of preterm m.1555A>G carriers within the next years.

## Conclusions

In this large cohort study of preterm infants with a birth weight below 1500 grams, frequency of the m.1555A>G mutation was 0.2% (12 of 7056 infants). M.1555A>G was associated with failed hearing screening at discharge if carriers were treated with aminoglycoside antibiotics. We found the mutation in all mothers of m.1555A>G-carriers. It is reassuring that large cohort studies in children and adults report normal hearing of m.1555A>G-carriers if they were not treated with aminoglycosides [2,6]. A British expert group recently suggested to screen all pregnant women for m.1555A>G to prevent aminoglycoside induced hearing loss in affected children [16]. Our data support such an approach – at least in women with preterm labour. However, it should be taken into account, that empiric aminoglycoside treatment of newborns and preterm infants with suspected sepsis is an extremely valuable treatment strategy and such screening programs should be carefully designed to avoid unwarranted replacement of established treatment schedules which include aminoglycoside antibiotics.

## Abbreviations

BPD: Bronchopulmonary dysplasia; CI: Confidence interval; CMV: Cytomegalovirus; DNA: Deoxyribonucleic acid; OR: Odds ratio.

## Competing interests

The authors declare that they have no competing interests.

## Authors’ contributions

WG conceived the study and drafted the manuscript, SB carried out the molecular genetic studies, MP and AZ performed the statistical analysis, HK, UFM, LG, MM, CH and EH participated in the design of the study, patient enrolment and helped to draft the manuscript. All authors read and approved the final manuscript.

## Pre-publication history

The pre-publication history for this paper can be accessed here:

http://www.biomedcentral.com/1471-2431/14/210/prepub
